# The Relationship between Motor, Imitation, and Early Social Communication Skills in Children with Autism 

**Published:** 2017-10

**Authors:** Hooshang Dadgar, Javad Alaghband Rad, Zahra Soleymani, Anahita Khorammi, Joe McCleery, Saman Maroufizadeh

**Affiliations:** 1 Department of Speech Therapy, School of Rehabilitation, Tehran University of Medical Sciences, Tehran, Iran.; 2 Department of Psychiatry, Roozbeh Psychiatric Hospital, Tehran University of Medical Sciences, Tehran, Iran.; 3 Roozbeh Hospital, Tehran University of Medical Sciences, Tehran, Iran.; 4 Center for Autism Research, Children’s Hospital of Philadelphia, Pyramid Educational Consultants, Philadelphia, USA.; 5 Department of Epidemiology and Reproductive Health, Reproductive Epidemiology Research Center, Royan Institute for Reproductive Biomedicine, ACECR, Tehran, Iran.

**Keywords:** *Autism Spectrum Disorder*, *Imitation*, *Motor Skills*, *Social Communication*

## Abstract

**Objective:** Development of early social skills in children is a complex process. To understand this process, it is important to assess how strengths or weaknesses in other developmental domains may be affected by these skills. The present study aimed at investigating the association of motor skills and imitation ability with early social communication skills in children with autism spectrum disorder (ASD).

**Method:** In this study, 20 children with ASD aged 3 to 5 years (M = 4.05, SD = 0.55) participated. All children were diagnosed as ASD based on the DSM-V criteria by an independent child psychiatrist. Additionally, Autism Diagnostic interview-Revised was used for subsequent diagnostic confirmation. Children were tested with Test of Gross Motor Development (TGMD-2), the Motor Imitation Scale (MIS), and the Early Social Communication Scales (ESCS). All examinations were videotaped for subsequent scoring. The relationship between these skills was estimated by Pearson correlation coefficient.

**Results: **A significant and strong correlation was obtained between TGMD total score and imitation total score (r =.776; p <0.001). However, the relationship between MIS subscales and TGMD-2 locomotor subtest scores was not significant (P>0.05). A significant correlation was found between MIS and TGMD total scores with Initiating Joint Attention and Responding to Joint Attention (p≤0/025) as ESCS subscales. But MIS and TGMD total scores were not correlated with social interaction and responding to behavioral requests subscales.

**Conclusion:** The results of the present study showed that indicated both imitation ability and motor function have an association with each other and with early social communication skills.

Autism Spectrum disorder (ASD) is a neurodevelopment disorder defined by impairment in social and communication skills as well as restricted behaviors and interest (1). Early social behaviors play a critical role in the acquisition of further social and language skills. In addition, social communication development in preschool years is associated with other developmental domains such as motor and cognition (2). For example, during the first years of life, acquisition of new motor ability provides opportunities for children to interact with objects and people (3).

On the other hand, a growing body of literature documented the important role of imitation in development of early language and social communication. Deficit in imitation skills has been widely studied in children with ASD (4). Several studies have reported that children with ASD did not differ from typical individuals in the imitation tasks. For example, Hobson and Hobson (5) noted that children with ASD have a similar ability in imitating goal-directed actions. However, there is consistent evidence suggesting that children with ASD have significant impairment in imitation skills (4).

Studies that focused on the relationship between imitation ability with these domains and those studies that examined the effect of imitation training on social communication have shown this critical role. Ingersoll and Schreibman (6) examined the efficacy of reciprocal imitation training on social communication skills in ASD. They reported that imitation training have positive effects on language, play, and joint attention in ASD.

To explain imitation deficit in ASD, various theories have been proposed such as identification theory (7), motor disturbance (8), social motivational hypothesis (9), and broken mirror neuron (10). Motor imitation skills are one of the common areas examined in studies of imitation impairment in ASD. Mostofsky, Dubey (11) found that children with ASD were significantly impaired in gestures and gestures with tool use. In addition, Williams, Whiten (4) in a review of studies on motor imitation in ASD, reported that even adolescents with ASD have problems in imitation. However, most studies have confirmed inability on motor imitation in these children. Motor execution problems have been proposed to account for some of these deficits in autism. However, some studies stated that praxis and dyspraxia deficits link to motor ability in ASD. Mostofsky and Dubey (11) reported that children with ASD performed less accurately in gesture imitation, gesture to command, and use of tool tasks, compared to typically developing children. They argued that a general praxis deficit might account for these problems. Although numerous studies have documented deficits in fine and gross motor skills in children with autism, few studies have considered the relationship between motor function and imitation in autism.

Given the theoretical importance of motor ability and findings of studies on typically developing children in development of language, several studies have focused on the association between motor and language skills in children with ASD. Some of these researches have focused on the predictive ability of early motor function for language development in siblings of children with ASD (12, 13). Gernsbacher and Sauer (14) found a significant association among early manual motor skills and later speech development in ASD. Pusponegoro and Efar (15) have also reported a positive relationship between gross motor and social skills in autism.

It is important to assess how strengths or weaknesses in each of these skills might be affected by the other. In addition, determining the relationship between each of these variables can be useful in early intervention planning. To summarize, prior studies have separately investigated the relationship between motor function, imitation abilities, and language skills. Therefore, the present study aimed at exploring the relationship between motor, imitation, and early social communication skills. We used early social communication scale (ESCS) to examine more communication components and identify the possible relationship between these variables.

## Materials and Methods

2.1, *Participants*

In this study, 20 children with ASD (18 males and 2 females), with the age range of 3 to 5 years (M = 4.05, SD = 0.55) participated. All children were diagnosed as ASD based on the DSM-V criteria by an independent child psychiatrist. Additionally, Autism Diagnostic interview-Revised (ADI-R) was used for subsequent diagnostic confirmation. Participants were recruited from 2 private rehabilitation centers and 3 speech therapy clinics. Inclusion criteria were as follow: (a) age 3 to 5 years, (b) absence of auditory or visual impairments, and (c) being free from motor and physical disabilities. The Ethic Committee of Tehran University of Medical Sciences approved the protocol study, and a written informed consent form was obtained from all parents prior to the participation of their children.

2.2, *Materials*

2.2.1, The Motor Imitation Scale (MIS; Stone, Ousley (16). 

The MIS was used to assess children’s imitation abilities. It consists of 8 objects and 8 gestures imitation tasks. In addition, half of the tasks was meaningful and the other half was not. The examiner displayed each imitation task to the children playfully and asked them to imitate. Each task was presented 3 times and responses were scored on 3 point scale for completeness of the imitation. Exact imitation received a score of 2, partial imitation a score of 1, and fail to imitate received a score of 0, yielding a possible range of 0 to 32. Only the best score was recorded for each item. The standardized alpha coefficient was .87, and test-retest reliability was .80 for the total MIS scores.

2.2.2. Test of Gross Motor Development (TGMD-2, Ulrich (17).

Motor skills were assessed using the TGMD-2. It was developed to assess gross motor skills in children aged 3 to 10 years. It includes 6 locomotor skills (run, gallop, leap, horizontal jump, hop, and slide) and 6 object control skills (striking a stationary ball, stationary dribble, kick, catch, and overhand throw, and underhand throw). Each skill is evaluated based on 3 to 5 performance criteria. Two trials are performed for each skill and scored 1, if the criteria are met, and 0 if the criteria are not met. Scores for locomotor and object control skills are obtained by summing the scores for related skills. Total TGMD-2 scores ranges from 0 to 48. Psychometric properties of the TGMD-2 were described by Ulrich (17).

2.2. 3The Early Social Communication Scales (ESCS; Mundy, Delgado (18).

The ESCS is a 20 minute structured and videotaped assessment used to investigate nonverbal communication skills in young children. The ESCS has been applied to assess 3 main behaviors including joint attention, behavioral requests, and social interaction in the experimenter and child’s interaction. Each behavior is also classified as an initiating or responding behavior. However, ESCS include initiating joint attention (IJA), responding to joint attention (RJA), initiating behavioral requests (IBR), responding to behavioral requests (RBR), initiating social interaction (ISI), and responding to social interaction (19). During the assessment, the child and experimenter seat at the table and the experimenter presents a series of toys and tasks and attempts to engage the child to elicit social-communication skills. Behavioral ratings are made from videotapes according to ESCS coding guidelines. Previous studies have shown good inter-rater reliability for the ESCS in normal and atypical children. 

2.3, *Procedures*

Parents of children were informed of the purpose and procedures of the study and were asked to complete a demographic questionnaire. Each child was evaluated individually in a quiet room on 2 separate sessions. A skilled speech therapist expert in teaching children with autism administrated all the assessments. At the beginning of each session, we asked the children to participate in a warm-up free play to acclimate them to the testing setting. The MIS and TGMD-2 were consecutively administrated at the first session and the ESCS was administrated at the second session. All examinations were videotaped for subsequent scoring.

*2.4, Data Analysis*

Statistical analysis was conducted using IBM SPSS Statistics for Windows, Version 22.0 (IBM Crop., Armonk, NY, USA). The Kolmogorov–Smirnov test was used to examine the normality of the data (p=0.651). Pearson correlation coefficient was used to evaluate the relationships between motor function, imitation skills, and early social communication skills. All statistical tests were two-sided, and a p<0.05 was considered statistically significant***.***

## Results

As displayed in Table 1, a significant strong correlation existed between TGMD total score and meaningful imitation (r = .871; p<0.001), non-meaningful imitation (r = .605; p=0.005), body imitation (r = .649; p = 0.002), and imitation total score (r = .776; p <0.001). The correlation between TGMD -2 object control subtest scores and MIS subscales was significant (P<0.05), but no statistically significant relationship was obtained between MIS subscales and TGMD-2 locomotor subtest scores (P>0.05).

Table 2 demonstrates the correlation between the ESCS subscales scores and TGMD, and MIS scores.

The results revealed a significant positive correlation between MIS total score and IJA (r = .737; <0.001), RJA (r = .728; <0.001), IBR(r = .688; p = 0.001). However, no significant correlation was found between MIS subscales and MIS total score with RBR, ISI, and RSI (p>0.05). TGMD-2 total score positively correlated with IJA (r = .849; <0.001), RJA (r = .826; <0.001), IBR (r = .603; p = 0.005), but did not correlate with RBR, ISI, and RSI (p>0.05). In addition, a significant correlation was obtained between TGMD-2 object control subtest with IJA (r = .838; <0.001), RJA (r = .831; <0.001) and IBR (r = .643; p=0.002), but not with RBR, ISI, and RSI. Finally, the correlation between TGMD-2 locomotor subtests with ESCS subscales was not significant (p>0.05).

**Table1:** Correlation Analysis between Imitation Skills and Motor Performance in Children With ASD (n = 20)

	**Locomotor**		**Object Control**		**TGMD Total Score**	
	*r*	*p*	*r*	*p*	*r*	*p*
Meaningful imitation	0.444	0.050	0.904	<0.001	0.871	<0.001
Non-meaningful imitation	0.204	0.389	0.662	0.001	0.605	0.005
Body imitation	0.266	0.258	0.695	0.001	0.649	0.002
Imitation total score	0.332	0.153	0.826	<0.001	0.776	<0.001

ASD: Autism Spectrum Disorders, TGMD: Test Of Gross Motor Development

**Table2:** Correlation Analysis between Imitation and Motor Skills with Early Social Communication Skills in Children with ASD (n= 20)

	IJA		RJA		IBR		RBR		ISI		RSI	
	*r*	*p*	*r*	*p*	*r*	*p*	*r*	*p*	*r*	*p*	*r*	*p*
**MIS total score**	0.737	<0.001	0.728	<0.001	0.688	0.001	0.357	0.122	0.435	0.055	0.232	0.325
Meaningful Imitation	0.781	<0.001	0.782	<0.001	0.683	0.001	0.334	0.150	0.398	0.082	0.252	0.283
Non- Meaningful Imitation	0.648	0.002	0.517	0.020	0.530	0.016	0.198	0.403	0.513	0.021	0.255	0.278
Body Imitation	0.616	0.004	0.651	0.002	0.661	0.002	0.409	0.073	0.328	0.158	0.181	0.446
**TGMD Total score**	0.849	<0.001	0.826	<0.001	0.603	0.005	0.368	0.111	0.319	0.171	0.015	0.950
Locomotor	0.562	0.010	0.500	0.025	0.253	0.282	0.177	0.454	0.214	0.365	-0.185	0.435
Object- control	0.838	<0.001	0.831	<0.001	0.643	0.002	0.385	0.094	0.314	0.178	0.079	0.742

IJA: Initiating Joint Attention, RJA: Responding to Joint Attention, IBR: Initiating Behavioral Requests, RBR: Responding to Behavioral Requests, ISI: Initiating Social Interaction, RSI: Responding to Social Interaction

## Discussion

The current study examined the relationship between motor function, imitation ability and social communication skills in children with ASD. Evidence exists on the connection between motor and other developmental domains, especially with cognition and language development. For example, Kim et al. (2016) suggested that the emergence of new motor skills provide an opportunity for the child to interact more with objects and people, causing an increase in social and language skills (2). Although the results showed these domains were closely related, initiation and response to social interaction were independent of motor and imitation skills. In addition, locomotor performance was not significantly related to social communication and imitation skills. Karasic Tamis-LeMonda and Adolph reported that the onset of walking in children increased locomotion towards their parents, thus, may facilitate their interaction (20). Our finding of a significant correlation between motor skills and imitation ability in ASD is consistent with that of the previous studies (11, 21). Zachor and Ilanit (22) compared imitation ability in an autism group with low and high level of fine and gross motor skills. They found that children with high scores on fine motor showed better performance on imitation tasks. In addition, support for these findings comes from studies, which related imitation deficits in ASD to praxis and motor execution problems. We found that performance on the imitation task was not related to locomotor, and this may be due to MIS tasks. MIS consists of 8 objects imitation tasks that focus on object use and may be more related to object control skills. In addition, in a study by Zachor and Ilanit (22), despite the relationship between fine motor and imitation, they found no significant correlation between gross motor and imitation skills in children with autism.

Our findings of a significant relationship between imitation ability and early social communication skills are in agreement with previous results. However, most of these studies have investigated the effect of imitation training on social communication, especially joint attention in ASD and have reported positive effects. No study has directly investigated the relationship between imitation and ESCS items in general. However, Ingersoll et al. have reported positive effects of imitation training on social communication behaviors such as joint attention, play, and language in several studies using different methods (6, 23, 24). Our finding that gross motor performances are related to joint attention is also consistent with that of other studies. Several studies have documented that the transition from crawling to walking leads children to actively engage in social interaction (25, 26). A study by Pusponegoro and Efar (15) indicated that children with autism and with gross motor impairment had low social skills. In a study by MacDonald and Lord (27), only object-control subscale was a predictor of social skills.

 In the current study, initiating social interaction and responding to social interaction were independent of imitation and gross motor. One possible explanation for this finding is social motivation theory and lack of motivation for social interaction. According to this hypothesis, lack of interest to social engagement in autism may be due to disruption in neural circuitry that is related to social motivation. Assaf and Hyatt (28), for example, have reported that motivational structures such as right Nucleus Accumbens (NAc) do not have a typical activity during reward-related motivation tasks in ASD. However, based on our knowledge, no study has used ESCS to determine a direct association with imitation, and lack of relationship might be due to differences in assessment tools.

## Limitation

This study had some limitations. The small study sample and the cross-sectional nature of the study did not allow us to draw any conclusions about the efficiency of imitation and motor skills as predictor variables on social communication. Furthermore, additional prospective longitudinal studies are needed to determine how these variables affect communication skills in ASD.

## Conclusion

In summary, according to the results of the current study, it can be concluded that both imitation ability and motor function have an association with some social skills. However, these results indicated that it could be beneficial for ASD to target imitation and motor skills in early intervention programs. Further studies must measure the effect of intervention protocols, especially motor function, on the communication skills in ASD.
